# Telomere length and COVID-19 disease severity: insights from hospitalized patients

**DOI:** 10.3389/fragi.2025.1577788

**Published:** 2025-06-10

**Authors:** Stijn Vos, Dries S. Martens, Elien De Waele, Geert Dewyspelaere, Geert Mistiaen, Pieter Goeminne, Tim S. Nawrot

**Affiliations:** ^1^ Centre for Environmental Sciences, Hasselt University, Hasselt, Belgium; ^2^ VITAZ hospital Sint-Niklaas, Sint-Niklaas, Belgium; ^3^ Department of Public Health & Primary Care, Occupational & Environmental Medicine, KU Leuven, Leuven, Belgium

**Keywords:** COVID-19 severity, respiratory health, telomere length, COVID-19, biological ageing

## Abstract

**Introduction:**

Telomere length is associated with various disease and immune function and may therefore impact COVID-19 disease severity. We studied the associations between telomere length as a geroprotective susceptibility marker and clinical outcomes in hospitalized COVID-19 patients.

**Methods:**

283 hospitalised COVID-19 patients (before vaccination, recruited between May 2020 and March 2021) were recruited for this cross-sectional study. Blood telomere length was determined by qPCR. The association between blood telomere length and clinical outcomes was examined using logistic regression, while adjusting for various covariates and confounders including demographic factors, comorbidity, body-mass index and blood cell counts. The primary clinical outcomes assessed were duration of stay, risk of ICU admission, and risk of requiring ventilation support.

**Results:**

Independent of sex and chronological age, an interquartile-range (IQR) increase in blood telomere length was associated with more favourable clinical outcomes in hospitalised COVID-19 patients: specifically, the odds ratio for ICU admission was 0.55 (95%CI: 0.32–0.88). Moreover, the odds ratio for the risk of ventilation was 0.52 (95%CI: 0.31–0.84). Finally, ordinal logistic regression revealed a lower odds for being in a higher quantile of hospital duration (OR: 0.79, 95%CI: 0.58–1.06).

**Discussion:**

To conclude, we found that in hospitalised COVID-19 patients, longer telomeres was associated with lower diseases severity in hospitalised COVID-19 patients, that could not be explained by shifts in blood cell counts. Therefore supporting the geroprotective or immunoprotective effects associated with longer telomeres conferring lower susceptibility to severe COVID-19 outcomes.

## Introduction

The SARS-CoV-2 virus, which causes the COVID-19 disease, has had a profound global impact on public health since its emergence in late 2019 (Ge et al., 2023; The Lancet Public Health, 2022; Haileamlak, 2021). The pandemic has not only placed large strain on healthcare systems worldwide (The Lancet Public Health, 2022; Haileamlak, 2021), but has also significantly affected economies, education, and daily life (Ge et al., 2023; Haileamlak, 2021). While most research has focused on the immediate effects of the virus, such as symptoms, transmission, and vaccine development (Ayouni et al., 2021), there is growing interest in the determinants of susceptibility for severe COVID-19 disease progression.

One emerging area of research is the potential relationship between COVID-19 and telomere length (Haridoss et al., 2023; Huang et al., 2022; Sanchez-Vazquez et al., 2021). Telomeres are the protective ends of chromosomes that shorten as cells divide. Studies have shown that external and environmental factors can influence telomere length (Francis et al., 2023; Howell et al., 2022; Martens et al., 2020; Martens et al., 2017; Shalev et al., 2013). When telomeres become too short, cells can no longer divide and become senescent or die. Telomere length has been associated with various health outcomes, including susceptibility to age-related diseases and cardiovascular outcomes (Huang et al., 2019; Blackburn et al., 2015; Gampawar et al., 2022; Nawrot et al., 2004; De Meyer et al., 2018). Telomere length is often considered a marker of biological aging and cellular senescence, which can be associated with age-related diseases and overall health (Huang et al., 2019; Blackburn et al., 2015; Gampawar et al., 2022; Nawrot et al., 2004; De Meyer et al., 2018). While not a geroprotective agent *per se*, telomere length can be indicative of geroprotective status. Longer telomeres are generally associated with healthier cells and a lower risk of age-related diseases, whereas shorter telomeres are linked higher risk of such diseases (Haycock et al., 2014). Therefore, longer telomere length can be viewed as a marker that suggests a more favourable ageing process, due to a lower susceptibility to age-related diseases and potentially a greater capacity for resilience against various stressors. However, it should be noted that higher telomere length is also associated with risk for certain cancers (Aviv et al., 2017; Savage, 2024), which highlights that telomere biology and its relation with ageing is a complicated topic. Nonetheless, telomere length can be considered a geroprotective marker, as it reflects the protective status against many adverse effects of ageing. Thus, telomere length may also be related to susceptibility to severe COVID-19 disease progression. However, it is important to note that telomere length itself is not an intervention or treatment but a biomarker that indicates the biological age and the potential health span of an individual.

There have been several studies investigating the link between viral infections and telomere length (Noppert et al., 2020; van de Berg et al., 2010; Wang et al., 2017). For instance, viral infections can cause inflammation and oxidative stress, which can accelerate telomere shortening (Wang et al., 2017; Gavia-García et al., 2021). However, the specific relationship between COVID-19 infection and telomere length is still not well understood.

Several studies suggest a potential link between COVID-19 severity and telomere length, with severe cases exhibiting shorter telomeres (Wang et al., 2021; Retuerto et al., 2022; Mongelli et al., 2021). Furthermore, research has indicated that both myeloid and lymphoid cells, key components of the immune response, are influenced by telomere length (Aviv, 2022), potentially impacting the body’s response to SARS-CoV-2, especially in older adults. One study has demonstrated that shorter telomere length has been associated with COVID-19 hospitalization and adverse outcomes, but not with clinical outcomes or post-COVID-19 manifestations (Retuerto et al., 2022). In COVID-19 survivors, a condition known as persistent post-COVID-19 syndrome (PPCS) can occur, and is associated with significant telomere shortening (Mongelli et al., 2021). These findings underscore the need for further research to fully understand the implications of telomere length on COVID-19 severity and aftermath.

In this study, our primary objective is to explore the potential association between COVID-19 and telomere length and genomic stability. To address this research question, we conducted a comprehensive analysis using blood samples collected from a cohort of hospitalized COVID-19 patients to predict the progression and severity of the disease, namely, duration of stay, risk of needing ventilation, and odds of admission to the intensive care unit (ICU).

## Materials and methods

### Study population

In total, we included 283 hospitalised patients with PCR-confirmed COVID-19 (Vos et al., 2023). 283 were recruited at time of admission to the hospital VITAZ (Sint-Niklaas, Flanders, Belgium): including 233 hospitalised at the general COVID-19 ward and 50 patients who required intensive care soon after admission. Patients were recruited between May 2020 and March 2021. To be eligible, patients had to be ≥18 years old, had to test positive for COVID-19 by PCR, had to not be included in other ongoing clinical intervention studies, and had to not have changed their residential address in the last 3 years. The participants enrolled in our study were not vaccinated at the time of the study. Based on information in the clinical records of the patients and the available information about dominant SARS-CoV-2 virus variant spread in Belgium and PCR tests, patients infected between May 2020 and 13 February 2021 were affected by the (original) Wuhan variant of the virus, while the majority of the patients recruited from 14 February 2021 until March 2021 were infected by the alfa variant of the virus. Written informed consent was obtained from all participants or their closest relatives and ethical approval was given by the ethical committee of VITAZ hospital and Hasselt University (Registration number: B2020115000006).

Demographic and clinical characteristics, such as ethnicity, sex, age, body mass index, smoking status (active, ex or never) and blood pressure on admission at the hospital were obtained from the medical records. Information on education was obtained by questionnaire. Educational attainment was defined as the highest educational level successfully completed using the International Standard Classification of Education (UNESCO, 2012). Patients educational level was coded as low, middle, and high. We chose not to ask participants about personal income because, based on experience in other population-based studies in Belgium, this question is often considered a violation of privacy (Martens et al., 2020; Nawrot et al., 2004). Blood samples were collected at admission to the ward. Subsequently, the values of more general biochemical and haematological measurements were determined at the time of admission (including C-reactive protein (CRP), absolute white blood cell count (WBC) and number of monocytes, eosinophils, lymphocytes, neutrophils, and platelets.

### Clinical outcomes

Primary clinical outcomes used in this study included the duration of hospitalisation (defined as the total number of days that patients remained hospitalised from the date of hospitalisation until the date of hospital discharge), admission to the intensive care unit, and necessity for invasive ventilation. We collected also data on parameters of comorbidity and determined the Charlson Comorbidity Index (Gasparini, 2018). Furthermore, early warning scores (EWS) (Royal College of Physicians, 2017; Kostakis et al., 2021) were determined at the time of admission to the hospital. The EWS is a scoring system which assists with the detection of changes in vital signs and may help to identify patients at risk for further clinical deterioration. The EWS is based on the following parameters: frequency of respiration, heart frequency, systolic blood pressure, consciousness, and body temperature, which makes it a relevant overall physiological indicator at the time of admission.

### Telomere length

DNA from whole blood, collected in BD Vacutainer^®^ plastic whole blood tubes with spray-coated K2EDTA (Becton, Dickinson), was extracted using the QIAamp DNA Mini Kit (Qiagen, Europe). Average relative telomere length was measured using a modified singleplex quantitative PCR (qPCR) method adapted from Cawthon, 2002 and 2009 (Cawthon, 2009; Cawthon, 2002). DNA integrity was assessed by agarose gel-electrophoresis. To ensure a uniform DNA input of 5 ng for each qPCR reaction, samples were diluted and checked using the Qubit™ dsDNA High Sensitivity Assay Kit (Life Technologies, Europe) using the Qubit™ Flex Fluorometer (Life Technologies, Europe). All samples were measured in triplicates on QuantStudio five real-time PCR system (Applied Biosystems) in a 384-well format. First, a single copy gene (human β globin) reaction was performed and this reaction mixture contained 5 ng DNA template, 1x KAPA SYBR^®^ FAST, Low ROXTM master mix (Kapa Biosystems, Merck) and 450 nM HBG1 primer (GCT​TCT​GAC​ACA​ACT​GTG​TTC​ACT​AGC) and 450 nM HBG2 primer (CAC​CAA​CTT​CAT​CCA​CGT​TCA​CC). Cycling conditions were as follows: one cycle at 95°C for 3 min, 40 cycles at 95°C for 3 s, and 58°C for 15 s. Second, a telomere-specific reaction was performed, containing 5 ng DNA template, 1x KAPA SYBR^®^ FAST, Low ROXTM master mix (Kapa Biosystems, Merck), 2 mM DTT, and 100 nM TelG primer (ACA​CTA​AGG​TTT​GGG​TTT​GGG​TTT​GGG​TTT​GGG​TTA​GTG​T) and 100 nM TelC primer (TGT​TAG​GTA​TCC​CTA​TCC​CTA​TCC​CTA​TCC​CTA​TCC​CTA​ACA). Cycling conditions were as follows: one cycle at 95°C for 3 min, two cycles at 94°C for 3 s and 49°C for 15 s, and 30 cycles at 94°C for 3 s, 62°C for 5 s, and 74°C for 10 s. After each qPCR a melting curve analysis was performed. On each run, PCR efficiency was evaluated using two standard six-point serial diluted standard curves (efficiencies were 105% for TL, 96% for HBG with an R2> 0.99 for all standard curves). Six inter-run calibrators (IRCs) were run to account for inter-run variability. The average relative TL were calculated using the qBasePlus 2.0 software (Biogazelle), and expressed as a calibrated normalized relative quantity (CNRQ). The latter is achieved by first calculating the RQ based on the delta-Cq method for telomere (T), and single-copy gene (S) obtained Cq values, using target specific amplification efficiencies. As the choice of a calibrator sample (sample to which subsequent normalization is performed, delta-delta-Cq) strongly influence the error on the final relative quantities (as a result of the measurement error on the calibrator sample), normalization is performed to the arithmetic mean quantification values for all analysed samples, which results in the NRQ. Samples are measured over different qPCR plates, therefore six IRCs are used to calculate an additional correction factor to eliminate run-to-run differences, resulting into the final T/S ratio (CNRQ). Mathematical calculation formulas to obtain RQ, NRQ and CNRQs are provided by Hellemans et al., 2007 (Hellemans et al., 2007). Our method precision is shown by an intra-assay ICC of 0.932 (95%CI 0.919 to 0.943; P < 0.0001). To test the repeatability of the assay, we extracted DNA for 10 patients DNA twice with a time interval of 3 months. TLs were measured in two independent runs and showed a high inter-assay ICC of 0.970 (95%CI 0.874 to 0.993; P < 0.0001). For 14 participants, relative telomere length could not be determined as the blood samples were not available or did not pass quality control.

### Statistical analysis

Statistical analyses were performed using R version 4.0.2 (R Core Team, Vienna, Austria). The threshold for statistical significance was set at the 95% confidence limit (α = 5%). Clinical outcomes investigated in this study included the duration of hospitalisation, risk of ICU admission, risk of need for invasive ventilation and early warning scores (EWS). Binomial logistic regression models were used to estimate the Odds Ratios (OR) for admission to the intensive care unit (ICU), and risk of ventilation. For duration of stay, ordinal logistic regression models were used, with duration of stay divided in to four quartiles as the ordinal outcome categories, as the linear model with telomere length and duration of stay did not pass linearity assumption check (as assessed by visual inspection of the residual plots). For analysis of early warning scores, we used multiple linear regression models.

We ran all models unadjusted, and we ran models only adjusted for age and sex (base models). All full models were adjusted for the following previously reported risk factors and potential confounders (selected *a priori*): age, sex, body-mass index (BMI), education, ethnicity, Charlson comorbidity score and smoking status. All model estimates reported in this study are calculated for an interquartile range (IQR) increase in relative telomere length.

Finally, we tested for potential effect modification by sex, age and virus variant by including the interaction term between relative telomere length and these factors in the full models.

## Results

### Study population

From May 2020 to March 2021, 283 hospitalised COVID-19 patients were recruited (Table 1). Patient age was, on average, 67.0 years old (range: 20.1–98.3). For patients admitted to the ICU, average age was 66.9 (range: 46.2–88.7), whereas for non-ICU patients, average age was 67.0 (range: 20.1–98.3).The study population included 127 (44.9%) women. In terms of comorbidities, 134 (47.3%) patients had congestive heart failure, 62 (21.9%) suffered from diabetes, and 62 (21.9%) participants had cancer. Most patients obtained a secondary education degree (n = 152, 53.7%), whereas 83 participants (29.3%) obtained a primary education degree or no degree at all and 48 participants (17.0%) obtained a college or university degree. A large proportion of the patients were of Caucasian ethnicity (n = 249, 88.0%). Patients with north-African ethnicity represented the second largest proportion (n = 21, 7.4%). Most patients never smoked (n = 172, 53.0%), whereas eight patients (2.8%) were active smokers.

**TABLE 1:** Description of the demographic and medical study population characteristics (n = 283).

	Mean (+- SD)/Median (25th– 75th pct)	Frequency (%)
Demographic characteristics		
Age (years)	67.0 (+- 16.5)	
BMI	27.9 (+- 5.5)	
Sex		
Male		156 (55.1%)
Ethnicity		
Caucasian		249 (88.0%)
North-African		21 (7.4%)
Middle-Eastern		6 (2.1%)
Asian		6 (2.1%)
Black-African		1 (0.4%)
Education		
Low		83 (29.3%)
Medium		152 (53.7%)
High		48 (17.0%)
Smoking status		
Active		8 (2.8%)
Ex		124 (43.8%)
Never		172 (53.0%)
Passive		1 (0.4%)
Blood cell counts		
Lymphocytes (count)	0.95 (0.70–1.26)	
Neutrophils (count)	4.61 (3.17–7.36)	
Eosinophils (count)	0.00 (0.00–0.01)	
Monocytes (count)	0.50 (0.40–0.80)	
Platelets (count)	201.00 (158.25–253.00)	
Outcomes		
Patients with congestive heart failure		134 (47.3%)
Patients with diabetes		62 (21.9%)
Patients with cancer		62 (21.9%)
Mortality (incident end points)		26 (9.2%)
Intensive care patients		50 (17.7%)
Patients requiring ventilation		48 (17.0%)
Duration of hospitalisation (days)	16.9 (+- 19.8)	
Charlson comorbidity index		
0		73 (25.8%)
1-2		105 (37.1%)
3-4		61 (21.6%)
≥5		44 (15.5%)

**FIGURE 1:**
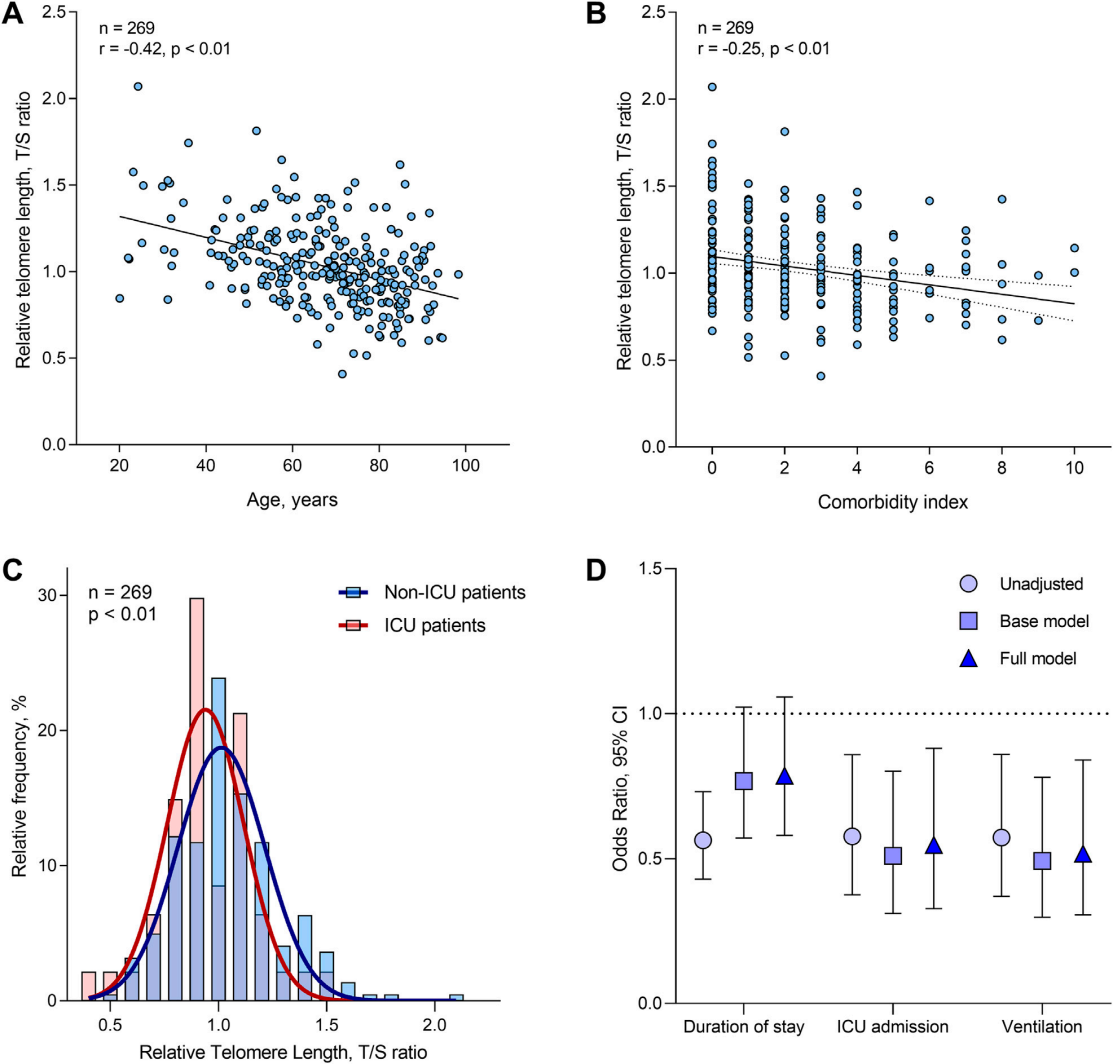
Distribution and determinants of telomere length in the study population, and associations with clinical outcomes. **(A)** Correlation between telomere length and chronological age. **(B)** Correlation between telomere length and Charlson comorbidity score. **(C)** Telomere length distribution in ICU versus non-ICU patients. **(D)** Associations between relative telomere length and COVID-19 clinical outcome (n = 269). Odds ratios are presented for an IQR increase in relative telomere length, and were determined by ordinal logistic regression (for duration of stay) or by binomial logistic regression (for risk of ICU admission and ventilation). Base models were adjusted for age and sex only. Full models were adjusted for age, sex, body-mass index (BMI), education, ethnicity, Charlson comorbidity score and smoking status. The brackets indicate the 95% confidence intervals.

Blood telomere length decreased by 0.61% (95% CI: 0.77% to −0.45%; r = −0.42, p < 0.01) for each year increase in age (Figure 1A), and did not differ between men and women (p = 0.89). BMI did not predict telomere length, with or without adjustment for chronological age (p = 0.09 and p = 0.84 respectively). Telomere length was 2.72% (95%CI: 3.95% to −1.48%) lower per point increase in the Charlson comorbidity score (Figure 1B). However, this association did not remain after adjusting for chronological age (p = 0.25). Finally, independent of age, telomere length was 10.12% (95%CI: 16.89% to −3.35%) lower for ICU patients as compared with non-ICU patients. The distribution of telomere length for both ICU and non-ICU patients is shown in Figure 1C.

### Clinical outcomes

The association between telomere length and clinical outcomes in COVID-19 hospitalised patients was examined using three models (Figure 1D): unadjusted; age and sex adjusted (base model); and adjusted for all covariates (full model). Furthermore, we performed sensitivity analyses, where we additionally adjusted our results for blood cell counts (neutrophils, eosinophils, monocytes, platelets and lymphocytes) (Table 2). The clinical outcomes of interest were duration of stay (divided into quantiles), risk of ICU admission, and risk of ventilation.

**TABLE 2:** Associations between relative telomere length and COVID-19 clinical outcome (n = 269).

Clinical outcome	Odds ratio	95% CI
Duration of stay		
Full model	0.79	0.58–1.06
+ Neutrophil counts only	0.80	0.59–1.09
+ All blood cell counts (neutrophils, eosinophils, monocytes, platelets and lymphocytes)	0.82	0.60–1.13
Risk of Ventilation		
N events/at risk (%)	48/269 (17.8%)	
Full model	0.52	0.31–0.84
+ Neutrophil counts only	0.43	0.23–0.73
+ All blood cell counts (neutrophils, eosinophils, monocytes, platelets and lymphocytes)	0.43	0.23–0.76
Risk of ICU Admission		
N events/at risk (%)	50/269 (18.6%)	
Full model	0.55	0.33–0.88
+ Neutrophil counts only	0.46	0.26–0.77
+ All blood cell counts (neutrophils, eosinophils, monocytes, platelets and lymphocytes)	0.48	0.26–0.82

Odds ratios are presented for an IQR, increase in relative telomere length, and were determined by ordinal logistic regression (for duration of stay) or by binomial logistic regression (for risk of ICU, admission and ventilation). Full models were adjusted for age, sex, body-mass index (BMI),education, ethnicity, Charlson comorbidity score and smoking status.

Duration of stay was divided into four quantiles based on the distribution of the outcome for analysis (first quantile: 1–6 days, second quantile: 6–9 days, third quantile: 9–14 days, fourth quantile: 14–113 days). By ordinal logistic regression, we calculated odds ratios and 95% confidence intervals that correspond with these quantiles of increased duration of stay. The risk for being in a shorter quantile for duration of stay decreased by 21% for an IQR increase in telomere length (OR: 0.79, 95% CI: 0.58–1.06) (Table 2) after adjusting for all covariates.

For risk of ICU admission, an IQR increase in telomere length was significantly associated with a lower risk both before, and after adjustment for covariates. The odds ratio of ICU admission was 0.55 (95% CI: 0.33–0.88). Similarly, the odds ratio for an IQR increase in relative telomere length associated with the risk of ventilation was 0.52 (0.31–0.84). In line with the previous analysis, we observed that an IQR increase in relative telomere length was associated with a 0.33 point decrease (95%CI: 0.64 to −0.03) in the early warning score, after adjusting for all covariates (Table 3).

**TABLE 3:** Associations between relative telomere length and early warning score (n = 269).

Model	Estimate	95% CI	p-value
Base model (age and sex only)	−0.30	−0.60–−0.01	0.05
Full model	−0.33	−0.64–−0.03	0.03
+ Neutrophil counts only	−0.40	−0.41–−0.08	0.01
+ All blood cell count (neutrophils, eosinophils, monocytes, platelets and lymphocytes)s	−0.41	−0.73–−0.09	0.01

Estimates are presented for an IQR, increase in relative telomere length, and were determined by multiple linear regression. Full models were adjusted for age, sex, body-mass index (BMI),education, ethnicity, Charlson comorbidity score and smoking status.

In the sensitivity analysis where we additionally adjusted for blood cell counts did not alter significantly the aforementioned associations (Table 2 and 3), with exception for duration of stay which was not significant.

### Interactions with sex, age and virus variant

We did not observe effect-modification of sex (p-value interaction ≥0.16), age (p-value interaction ≥0.23), or virus variant (p-value interaction ≥0.24) on telomere length for any of the studied outcomes.

## Discussion

The main finding of this study was an association between clinical outcomes (risk of ICU admission and ventilation) in hospitalised COVID-19 patients and relative telomere length as determined in their blood at the time of hospital admission. We found a greater resilience against COVID-19 disease progression associated with longer telomere length in hospitalised patients. This finding aligns with some existing literature, but contrasts with others. For instance, a study found a strong inverse correlation between relative leukocyte telomere length (LTL) and COVID-19 severity (Mahmoodpoor et al., 2023). This suggests that telomere length could potentially be linked to the severity of the disease, which in turn could influence the duration of hospital stay. However, a systematic review and meta-analysis found no clear evidence for an association between shorter telomere length and severe COVID-19 disease (Haridoss et al., 2023). These conflicting results in the literature highlight the complexity of the relationship between telomere length and COVID-19 outcomes. Further research is needed to clarify this relationship and to understand the underlying mechanisms, particularly studies that account for important factors such as blood cell counts, age and sex. More research on the associations between telomere length and Long-COVID may be of interest as well (Polli et al., 2025). Our study contributes to this ongoing investigation by providing evidence of an association between COVID-19 hospital stay duration and telomere length independent of shifts in blood cell count.

The biological mechanisms linking telomere length and COVID-19 severity are complex and multifaceted. Telomeres, located at the ends of chromosomes, play a crucial role in cell proliferation and immune competency. Shortened telomeres could impede tissue regeneration, potentially exacerbating the severity of COVID-19. Furthermore, DNA damage signalling induced by ageing telomeres increases the expression of ACE2, the human SARS-CoV-2 cell receptor (Sepe et al., 2022). This could potentially enhance the susceptibility of cells to SARS-CoV-2 infection, leading to more severe disease outcomes. Additionally, lymphopenia, a common feature of severe COVID-19, is associated with a significant reduction in T cell count, which has been linked to poor prognosis in patients (Chen et al., 2023). This is related to telomere length biology, since shorter TL means impeded T-cell proliferation (Aviv, 2020). Furthermore, telomere length has been found to have a significant association with immune function, as a genetic causal relationship between shorter and compromised immune cell function has been shown (Li et al., 2024). For instance, a positive correlation was found between telomere length and certain immune cells such as CD28, CD45RA and the percentage of CD8br cells within the CD8^+^ cell population, while a negative correlation was found with others such as Transitional AC (Li et al., 2024). These findings suggest a link between immune function and telomere length, which may explain why COVID-19 disease severity is associated with telomere length in hospitalised patients, as demonstrated in this study. Finally, research indicates that telomere length is vital for T-cell clonal expansion and its decline with age may contribute to higher COVID-19 mortality (Anderson et al., 2022). Individuals maintain a robust T-cell replication capacity until around the sixth decade, after which a rapid decline in this capacity coincides with an increase in COVID-19 deaths (Anderson et al., 2022). A higher proportion of short T-cell telomeres was associated with lower lymphocyte counts, suggesting that telomere erosion may underlie the lymphopenia observed in severe cases (Kraft et al., 2024). Similarly, analyses in elderly patients confirmed that the buildup of critically short telomeres is linked to compromised T-cell proliferative responses, underscoring that both aging and inherent telomere shortening can diminish immune defences (Benetos et al., 2021).

Alternatively, besides being used as a biomarker for vulnerability to COVID-19, there is also studies showing how telomere dynamics may be affected by COVID-19. COVID-19 is associated with accelerated telomere attrition (Cao et al., 2022), as indicated by DNAmTL (Lu et al., 2019) measurements. Deceased COVID-19 patients experienced accelerated telomere attrition, with a significantly different dynamic attrition pattern compared to patients who recovered (Bejaoui et al., 2023).

We recognize the limitations of using LTL as a conventional biomarker of ageing (Vaiserman and Krasnienkov, 2020; Aviv and Shay, 2018). Despite this, our study suggests that TL can be a valuable indicator of susceptibility to certain age-related diseases, in this case severe COVID-19 disease. It’s crucial to understand that while LTL may not fully reflect biological age, telomere length at birth, combined with the rate of telomere shortening observed over the course of life (Martens et al., 2021), provides significant insights into biological vulnerability to specific diseases (Schneider et al., 2022). For instance, studies have found a correlation between both shorter and longer TL and higher cancer risk (Aviv et al., 2017; Savage, 2024), while shorter TL is often linked to atherosclerotic cardiovascular diseases (Haycock et al., 2014; Zhan and Hägg, 2019). Therefore, although TL may not act as a traditional biomarker of ageing, as a biological marker it possesses considerable predictive value for age-related disease susceptibility and longevity in adults This highlights the continued critical role of TL in ageing research.

Our study has several strengths. Firstly, it is one of the few studies investigating the association between telomere length and COVID-19 severity, contributing to a relatively unexplored field of research. Secondly, the use of hospital duration as a proxy for COVID-19 severity provides a quantifiable and objective measure. Additionally, we were able to adjust our findings not only for factors such as sex and chronological age, but also for blood cell counts. This is important, since we measured telomere length in blood collected after the time of hospital admission. Therefore, telomere length may have been affected due to differences in blood cell composition. However, our study also has limitations. The sample size, while adequate, could be larger to increase the statistical power and generalizability of our findings. Additionally, the measurement of telomere length in blood may not fully capture the telomere dynamics in tissues directly affected by SARS-CoV-2. Furthermore, we did not had objective measures of immune cell function to understand better the underlying mechanisms.

## Conclusion

These findings suggest that telomere length predicts disease severity in COVID-19 patients. The observed associations between telomere length and better clinical outcomes highlight the potential importance of telomere biology in the context of COVID-19. Further research should elucidate the geroprotective or immunoprotective effects accompanied with longer telomeres as a possible mechanism conferring the higher resilience to severe COVID-19 progression.

## Data Availability

The raw data supporting the conclusions of this article will be made available by the authors, without undue reservation.
